# HIV testing in patients who are HCV positive: Compliance with CDC guidelines in a large healthcare system

**DOI:** 10.1371/journal.pone.0252412

**Published:** 2021-06-02

**Authors:** Ilan Fleisher, Alexander G. Geboy, Whitney Nichols, Sameer Desale, Stephen Fernandez, Peter Basch, Dawn A. Fishbein

**Affiliations:** 1 MedStar Health Research Institute, MedStar Health, Hyattsville, MD, United States of America; 2 New York Medical College, Valhalla, NY, United States of America; 3 Association of American Medical Colleges, Washington, DC, United States of America; 4 Frank H. Netter School of Medicine at Quinnipiac, North Haven, CT, United States of America; 5 MedStar Health, Columbia, MD, United States of America; 6 MedStar Washington Hospital Center, Washington, DC, United States of America; East Carolina University Brody School of Medicine, UNITED STATES

## Abstract

**Background:**

There are approximately 300,000 people in the United States who are co-infected with HIV and HCV. Several organizations recommend that individuals who are HCV infected, as well as persons over the age of 13, should be HIV tested. Comorbidities associated with HCV can be reduced with early identification of HIV. Our objective was to determine whether providers routinely followed HIV testing guidelines for patients who tested HCV positive (HCV+).

**Methods:**

A retrospective chart review was conducted of all patients in primary care at an academic health system from 7/2015–3/2017 who tested HCV+. As part of a primary database, HCV testing data was collected; HIV testing data was abstracted manually. We collected and described the intervals between HCV and HIV tests. To determine associations with HIV testing univariable and multivariable analyses were performed.

**Results:**

We identified 445 patients who tested HCV+: 56.6% were tested for HIV, the mean age was 57 ± 10.9 years, 77% were from the Birth Cohort born 1945–1965 (BC); 61% were male; and 51% were Black/AA. Patients in the BC were more likely to be HIV tested if they were: male (p = 0.019), Black/AA (p<0.001), and had Medicaid (p = 0.005). These differences were not found in the non-BC. Six patients who were tested for both HIV and HCV were found to be newly HIV positive at the time of testing.

**Conclusion:**

As demonstrated, providers did not routinely follow CDC recommendations as almost half of the HCV+ patients were not correctly tested for HIV. It is important to emphasize that six persons were tested HIV positive simultaneously with their HCV+ diagnosis. If providers did not follow the CDC guidelines, then these patients may not have been identified. Improvements in EHR clinical decision support tools and provider education can help improve the HIV testing rate among individuals who are HCV+.

## Introduction

There are approximately 300,000 people in the US who are co-infected with HIV and HCV [1] and estimated at 2.75 million worldwide [2]. The Centers for Disease Control and Prevention (CDC) in the United States (US) recommends that persons with HCV should be tested for HIV, even if previously HIV negative [3] and the CDC recommends all persons over the age of 13 be HIV tested, at least once [4]. Studies conducted in the US have shown that patients within single hospitals or the Veteran Health Administration (VA) have not been adequately tested for HIV given their coinfection with HCV [5,6]. These latter studies were conducted prior to HIV universal and HCV Birth Cohort screening recommendations [4,7]–thus screening should now be even more prevalent. The Birth Cohort, sometimes referred to as the “Baby Boomers” includes all individuals born between 1945–1965. This age group has been found to have the highest prevalence of HCV due to risks during that era. Thus the CDC recommend that all those in the Birth Cohort receive at least a one HCV antibody test in their lifetime [8]; this was prior to the more recent universal HCV testing recommendations by the USPSTF to test all individuals between the ages of 18–79 [9]. Studies similar to the above from the US [5,6] were conducted in the United Kingdom–and even with the same HIV testing recommendations from the National Institute for Health and Care Excellence as the US CDC, they still found that up to 55% of persons with HCV have not been correctly tested for HIV [10].

To our knowledge, a study looking at HIV testing in HCV positive patients has not been conducted at a large regional healthcare corporation. Importantly, Lin et al (2013) reported on the accelerated pathogenesis of liver fibrosis in patients who are co-infected with HIV and HCV [11]. As HCV is now widely curable with the advent of new direct-acting antiviral agents, early identification and treatment in patients with HIV is crucial in order to appropriately manage liver disease. Identifying people with HIV also allows providers to prevent HIV transmission and initiate treatment earlier. With the broadening of CDC and USPSTF HCV antibody screening guidelines to include those in the Birth Cohort [12] and more recently anyone 18–79 years of age [10], there is an opportunity to enhance provider awareness for HIV screening. Our objective was to determine whether CDC guidelines for HIV testing were routinely followed in HCV and primary care clinical settings at a large healthcare system when patients test HCV positive (HCV+). We hypothesized that HIV was not tested for within guidelines.

## Methods

We conducted a retrospective chart review for patients seen in primary care and HCV settings at MedStar Health, a large regional healthcare system, from July 2015 through March 2017. MedStar Health is the largest regional healthcare system in the Baltimore-Washington DC Metropolitan area, consisting of 10 major hospitals and operating over 200 entities in the region. A convenience sample within MedStar Health was taken and included all persons who were > 18 years, who were tested for HCV and newly tested HCV+, within the study timeframe. A waiver of consent and HIPAA was obtained from the MedStar Health Research Institute (MHRI) Institutional Review Board for this study. Data for the patient cohort was collected by the MHRI Biomedical Informatics Department through the Cerner MedConnect electronic health record (EHR) and Explorys data systems and then stored and uploaded in a password protected Excel file. Data remained identified to allow for a manual chart review and abstraction of data from the EHR.

We categorized the HCV testing data into patients born in the Birth Cohort (“BC”) and the non-Birth Cohort (“non-BC”), and we included gender, race/ethnicity, HCV antibody (Ab) status and testing dates, provider type, clinical outcomes and insurance. Patients were classified between the BC and non-BC to determine whether there were any differences in HIV testing given the prior CDC and USPSTF BC HCV screening guidelines published prior to this study. Insurance type included Medicaid, Medicare or private insurance. Patients may either have Medicaid or Medicare depending on age, co-existing morbidities or if they have an income below a certain level.

Determining the HIV Ab testing status (tested, yes/no) of each patient was abstracted manually as results done prior to mid-2016 were recorded in text format due to an EHR conversion and data migration. All serologic versions of the HCV and HIV Ab tests were included. One researcher, IF, conducted the manual chart review of HCV+ patients to determine their HIV testing status and date of test, which were recorded in the Excel spreadsheet provided by MHRI Biomedical Informatics. A random audit of the data abstraction was performed by other study members and discrepancies were reviewed with final determination by study PI (DF). We reviewed the entire patient’s chart to make sure that they were not tested for HIV prior to their HCV testing. Similarly, it was noted whether the HIV test was performed in conjunction with the first HCV test. Patients who were seen in care after their initial HCV+ result, may subsequently have had HIV testing along with their treatment. This allowed us to note which provider ordered both tests and determine if this was completed by the same or different providers. We followed up with patient charts three months after the last inclusion date to allow for providers to test for HIV within a reasonable time after the initial appointment. If patients tested positive for both HIV and HCV, then their chart was reviewed even further to determine whether it was their first time receiving this coinfection diagnosis. Using the dates for HIV and HCV testing, the interval between HCV and HIV Ab testing was assessed and described to determine differences in the date of HIV and HCV testing; and the time interval if they were tested on different dates. Patient charts were reviewed multiple times during study follow up to determine if a subsequent HIV test was performed for patients without any prior history of HCV testing.

Univariable and multivariable analyses were performed using www.vassarstats.net and R version 3.6.1, respectively to determine associations with HIV testing. P-values were considered statistically significant when less than 0.05. Associations between categorical variables were conducted using Yates chi-squared or Fisher’s exact tests, the latter for small cell sizes. A comparison of means for continuous variables was conducted using two sample t- or non-parametric Wilcoxon rank sum tests, the latter for non-normally distributed variables. Statistical analyses were conducted for the entire cohort; subgroup analyses included comparisons within the BC and non-BC cohorts. We compared HIV testing for the entire cohort and within each stratified age cohort (BC and non-BC), race/ethnicity, gender, and insurance type by univariable analysis. We applied multivariable logistical regression for the entire cohort to assess for an independent association of predictors for HIV testing (dependent categorical variable) in the categorical age cohorts (BC and non-BC) while adjusting for race/ethnicity, gender, and insurance type. Variables were included in the multivariable model if they had a p-value of less than or equal to 0.05. Multicollinearity was examined by calculating variance inflation factor (VIF).

## Results

**Fig 1:**
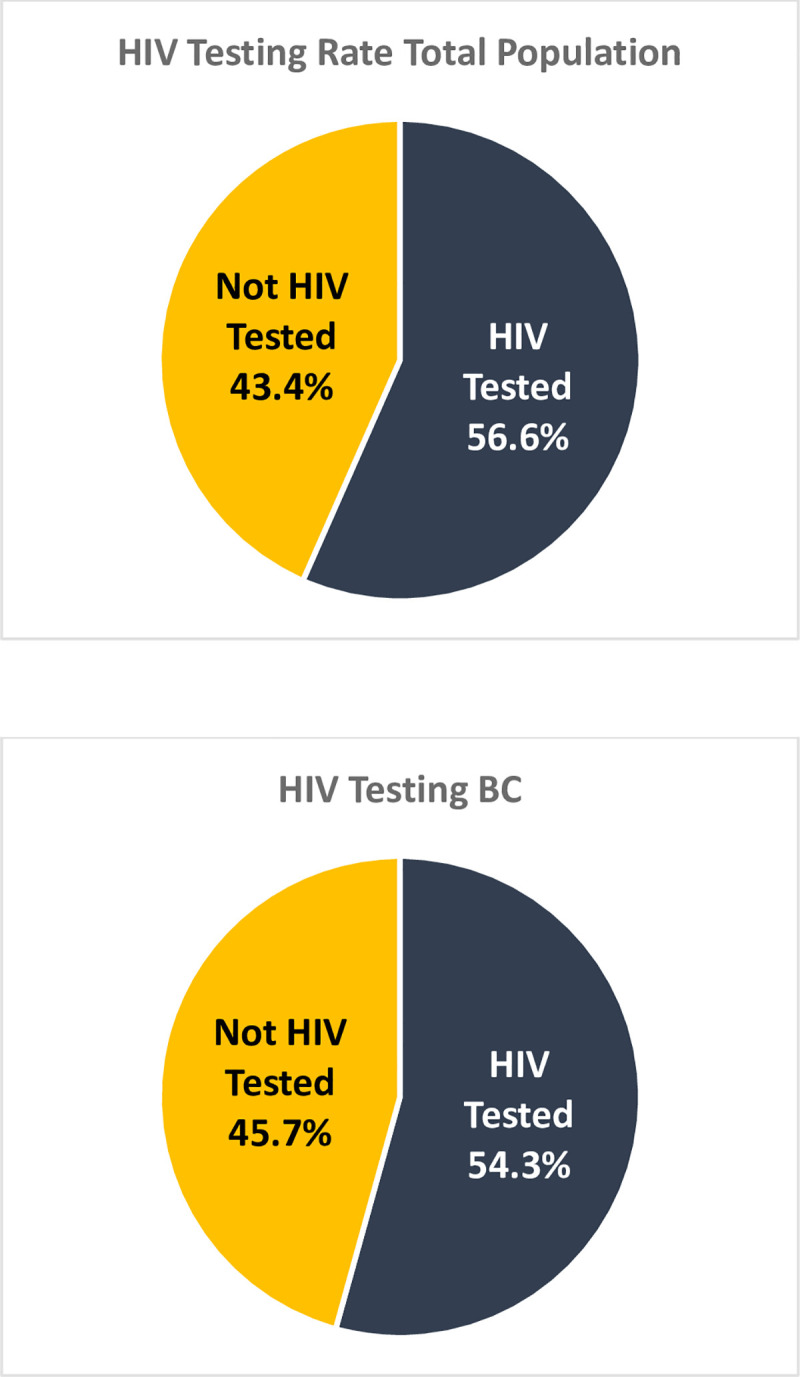
HIV testing in the HCV Ab+ population. This figure illustrates the rates of HIV testing rates for those in BC, non-BC, and study as a whole. Patients were not more likely to be tested for HIV is they were in the non-BC compared to the BC (p = 0.09).

There were 445 HCV+ patients tested and identified during this period (**Table 1**): the mean age was 57 ± 10.9 years; 76.6% (n = 341) were in the BC, 61.1% (n = 272) were male, 51.0% (n = 227) were Black/African American (B/AA) and 36.0% (n = 160) were White. During this period, 56.6% (n = 252) of all HCV+ persons, 54.3% (n = 185) of BC patients and 64.4% (n = 67) of non-BC patients, were tested for HIV at any (**Fig 1**); 67.5% (n = 170) of HIV tests were ordered simultaneously with the HCV Ab tests. Six patients (2.4% of all HIV tested) were newly positive for both HIV and HCV, and all six tests were conducted simultaneously with HCV testing. There were significantly fewer days between the HCV and HIV Ab tests if they were ordered by the same provider, rather than different providers (p<0.0001).

**Table 1:** Patient baseline demographics.

	Birth Cohort (BC) (N = 341)	Non-Birth Cohort (non-BC) (N = 104)	Total (445)
Age (Mean ± SD)	60.8 ± 5.0	Above BC: 76.1 ± 2.3 Below BC: 39.5 ± 9.2	57.2 ± 10.9
Sex			
Female	127 (37.2%)	46 (44.2%)	173 (38.9%)
Male	214 (62.8%)	58 (55.8%)	272 (61.1%)
Race			
Black/AA	191 (56.0%)	36 (34.6%)	227 (51.0%)
White	105 (30.8%)	55 (52.9%)	160 (36.0%)
Other	45 (13.2%)	13 (12.5%)	58 (13.0%)
Ethnicity			
Hispanic	5 (1.5%)	3 (2.9%)	8 (1.8%)
Not Hispanic	324 (95.0%)	97 (93.3%)	421 (94.6%)
Other	12 (3.5%)	4 (3.8%)	16 (3.6%)
Insurance Type			
Public			
Medicaid	130 (38.6%)	49 (47.6%)	179 (40.2%)
Medicare	69 (20.5%)	15 (14.6%)	84 (18.9%)
Private	138 (40.9%)	39 (37.9%)	177 (39.8%)

This table includes the basic demographic information for the patients involved in this research study.

On univariable analysis for the entire cohort (**Table 2**), patients were more likely to be HIV tested if they were: male (p = 0.04), B/AA (p = 0.002), and had Medicaid insurance (p = 0.004). There were more patients who were HIV tested in the non-BC (64.4%) than the BC (54.3%) (p = 0.09), though this did not reach statistical significance. Within the BC, patients were more likely to be HIV tested if they were: male (p = 0.019), B/AA (p<0.001), and had Medicaid insurance (p = 0.005) (**Table 3)**. In the non-BC, there were no statistically significant differences in the likelihood of HIV testing between gender, race/ethnicity or insurance type (**Table 3)**. On multivariable analysis for the entire cohort, patients were more likely to be HIV tested: in the non-BC v the BC (OR_adj_ 1.84, CI_95_ 1.14–3.01); were B/AA (2.25, 1.46–3.50) and other (2.25, 1.20–4.30) as compared to White; were male (1.63, 1.10–2.44) and had Medicaid insurance (1.77, 1.18–2.65) (**Table 4**). VIF was less than two for all variables included in the multivariable analysis, depicting no multicollinearity.

**Table 2:** Univariable analysis of HIV testing rates in the entire cohort.

Entire Cohort					
	Not HIV Tested n(%)	HIV Tested n(%)	OR	95% Confidence Interval	p-value
Sex					
Female	86(50)	87(5)	0.66	0.45–0.96	**p = 0.04**
Male	107(39)	165(61)	Ref		
Race					
Balck/AA	85(37)	142(63)	1.94	1.29–2.93	**p = 0.002**
White	86(54)	74(46)	Ref		
Other	22(38)	36(62)	1.90	1.03–3.52	p = 0.056
Ethnicity					
Hispanic	4(50)	4(50)	Ref		
Not Hispanic	183(43)	238(57)	1.30	0.32–5.27	p = 0.73
Other	6(37)	10(63)	1.67	0.30–9.27	p = 0.67
Insurance					
Medicaid	62(35)	117(65)	1.70	1.11–2.61	**p = 0.019**
Medicare	44(52)	40(48)	0.82	0.49–1.38	p = 0.54
Private	84(47)	93(52.5)	Ref		
Medicaid	62(35)	117(65)	2.07	1.23–3.52	**p = 0.009**
Medicare	44(52)	40(47.6)	Ref		
Medicaid	62(35)	117(65)	1.82	1.23–2.69	**p = 0.004**
NonMedicaid*	128(49)	133(51)	Ref		

This table includes univariable p-values, odd ratios, and confidence intervals for the entire cohort of this research study.

*NonMedicaid = Medicare + Private Insurance.

**Table 3:** Univariable analysis of HIV testing rates in the BC and Non-BC.

	Birth Cohort (BC)					Non-Birth Cohort (non-BC)				
	Not HIV Tested n(%)	HIV Tested n(%)	OR	95% Confidence Interval	p-value	Not HIV Tested n(%)	HIV Tested n(%)	OR	95% Confidence Interval	p-value
Sex										
Female	69 (54)	58 (46)	0.57	0.37–0.80	**p = 0.019**	17 (37)	29 (63)	0.90	0.40–2.01	p = 1.00
Male	87 (41)	127(59)	Ref			20 (35)	38 (65)	Ref		
Race										
Black/AA	72(38)	119(62)	2.80	1.71–4.58	**p<0.001**	13 (36)	23 64)	1.75	0.42–2.42	p = 0.84
White	66(63)	39(37)	Ref			20(36)	35(63)	Ref		
Other	18(40)	27(60)	2.54	1.24–5.19	**p = 0.016**	4(31)	9(69)	1.28	0.35–4.72	p = 0.76
Ethnicity										
Hispanic	2(40)	3(60)	Ref			2(67)	1(33)	Ref		
Not Hispanic	148(46)	176(54)	0.79	0.13–4.81	p = 1.00	35(36)	62(64)	3.54	0.31–40.49	p = 0.55
Other	6(50)	6(50)	0.67	0.08–5.54	p = 1.00	0(0)	4(100)	n/a		
Insurance										
Medicaid	46(35)	84(64)	1.93	1.18–3.16	**p = 0.01**	16(33)	33(67)	1.03	0.42–2.52	p = 0.86
Medicare	36(52)	33(48)	0.97	0.54–1.73	p = 1.00	8(53)	7(47)	0.43	0.13–1.47	p = 0.30
Private	71(51)	67(49)	Ref			13(33)	26(67)	Ref		
Medicaid	46(35)	84(65)	1.99	1.10–3.61	**p = 0.033**	16(33)	33(67)	2.36	0.73–7.65	p = 0.25
Medicare	36(52)	33(48)	Ref			8(53)	7(47)	Ref		
Medicaid	46(35)	84(65)	1.95	1.24–3.07	**p = 0.005**	16(33)	33(67)	1.31	0.58–2.95	p-0.65
NonMedicaid*	107(52)	100(48)	Ref			21(39)	33(61)	Ref		

This table includes univariable p-values, odd ratios, and confidence intervals for the BC and non-BC for this research study.

*Not Medicaid = Medicare + Private Insurance.

**Table 4:** Multivariable analysis: Association with HIV testing.

	OR	95% Confidence Interval	P value
Cohort Non-Birth Cohort	1.84	1.143–3.007	0.013
Ref- Birth Cohort			
Race Black/AA	2.25	1.463–3.496	< 0.001
Race Other	2.25	1.204–4.302	0.012
Ref—White			
Sex Male	1.63	1.096–2.442	0.016
Ref—Female			
Insurance Type: Medicaid	1.77	1.182–2.650	0.006
Ref- Insurance Type: NonMedicaid*			

This table includes a multivariable analysis of independent predictors for HIV testing for the entire cohort.

* NonMedicaid = Medicare + Private Insurance.

## Discussion

As demonstrated above, nearly one-half (43.4%) of persons with an HCV+ test were *not* HIV tested, nor were they ever tested during this study period. Although all persons between 13–64 and anyone with HIV specific risk factors should be HIV tested [4], we need to emphasize the importance of testing in patients who are HCV+, as six persons were newly HIV positive in concurrence with their positive HCV diagnosis. If providers were not following the HIV testing guidelines prior to HCV testing, then these individuals may not have been diagnosed with HIV in a timely manner. Our analysis was conducted to ascertain which groups of people might be more or less likely to receive HIV screening in accordance with CDC guidelines.

Our data show that there may be certain demographics which were more likely to have HIV testing including those who were in the non-BC, Black/AA, male and who had Medicaid insurance. This data was shown to hold true in both the univariable analysis and multivariable logistical regression. There might be a higher perceived or actual risk for patients in these groups, which trigger providers to test these groups more regularly for HIV. One study showed that US patients who identified as Hispanic or B/AA were tested for HIV at higher rates than those who identified as White [13]. Similarly, HIV testing rates were higher when the visit was conducted at a metropolitan area compared with a nonmetropolitan area [13]. Our data also confirms the CDC surveillance findings that the groups most likely to be tested in the US are those with the highest perceived risk, being B/AA males [14]. To our knowledge, we could not find other studies showing that persons with Medicaid insurance is the driver for HIV testing in patients with HCV, as we discovered in our cohort. Our results also align with similar studies in other settings that show that there is insufficient testing for HIV in HCV infected patients [5,6]. Not only is there inadequate testing in single hospitals and in the VA, but this is also an issue in this large regional healthcare system, and likely others as well.

### Limitations

There are a few limitations for this study. We could not ascertain whether these persons with HCV were seeking treatment for HCV or HIV. It is possible that patients were seeking treatment for potential HIV and happened to get an HCV test, not the other way around. Similarly, we did not have access to patient records outside our health system. If a patient had their HCV Ab testing done within the system and then decided to go elsewhere for their HCV care where they subsequently had an HIV test, then we did not know whether this test was performed, unless the information was sent explicitly to their provider and documented. An additional limitation includes those inherent in a retrospective design including the use of convenience sampling and the possibility of confounding variables. In our study it is possible that there are other variables that are associated with the outcome that we were unaware of during this study.

### Future directions

Future research studies should focus on determining the best methods to increase the rate of HIV testing in the primary care setting. Improvements in provider education and electronic health record (EHR) clinical decision support tools may be useful and warranted in order to appropriately test patients for HIV. These enhancements could allow for increased identification and engagement in care of those who are HIV and HCV co-infected, and consequently improvement in health outcomes.

## Conclusions

Despite both universal HIV recommendations and those recommended in persons with HCV, a high proportion of persons were not tested for HIV. Improvements in provider education and EHR clinical decision support tools will be beneficial to help appropriately administer HIV tests for patients who are HCV+, as well as all patients. This notification should alert the provider in the patient’s chart for patients who have not previously tested positive for HIV, especially when they have a positive HCV antibody test. We hope that if providers follow these guidelines, we are able to identify potential patients with HIV earlier in their disease stage, and so that we may more effectively care for them.

## Supporting information

S1 File HIV testing de-identified PLOS One submission. (XLSX)
